# Real‐world evidence of efficacy of pembrolizumab plus chemotherapy and nivolumab plus ipilimumab plus chemotherapy as initial treatment for advanced non‐small cell lung cancer

**DOI:** 10.1111/1759-7714.15304

**Published:** 2024-04-11

**Authors:** Ayami Kaneko, Nobuaki Kobayashi, Kenji Miura, Hiromi Matsumoto, Kohei Somekawa, Tomofumi Hirose, Yukihito Kajita, Anna Tanaka, Shuhei Teranishi, Yu Sairenji, Hidetoshi Kawashima, Kentaro Yumoto, Toshinori Tsukahara, Nobuhiko Fukuda, Ryuichi Nishihira, Keisuke Watanabe, Nobuyuki Horita, Yu Hara, Makoto Kudo, Naoki Miyazawa, Takeshi Kaneko

**Affiliations:** ^1^ Department of Pulmonology Yokohama City University Graduate School of Medicine Yokohama Japan; ^2^ Department of Respiratory Medicine Yokohama Sakae Kyosai Hospital Yokohama Japan; ^3^ Department of Pulmonology Yokohama City University Medical Center Yokohama Japan; ^4^ Department of Respiratory Medicine Kanto Rosai Hospital Kawasaki Japan; ^5^ Department of Respiratory Medicine Yokohama Minami Kyosai Hospital Ykohama Japan; ^6^ Department of Respiratory Medicine Chigasaki Municipal Hospital Chigasaki Japan; ^7^ Department of Respiratory Medicine Fujisawa Municipal Hospital Fujisawa Japan; ^8^ Department of Respiratory Medicine Yokohama Nanbu Hospital Yokohama Japan

**Keywords:** ipilimumab, nivolumab, non‐small cell lung cancer, pembrolizumab, real‐world evidence

## Abstract

**Background:**

For advanced non‐small cell lung cancer (NSCLC), combination therapies including a PD‐1 inhibitor plus chemotherapy or a PD‐1 inhibitor, CTLA‐4 inhibitor, and chemotherapy are standard first‐line options. However, data directly comparing these regimens are lacking. This study compared the efficacy of pembrolizumab plus chemotherapy (CP) against nivolumab plus ipilimumab and chemotherapy (CNI) in a real‐world setting.

**Methods:**

In this multicenter retrospective study, we compared the efficacy and safety of CP and CNI as first‐line therapies in 182 patients with stage IIIB–IV NSCLC. Primary outcomes were overall survival (OS) and progression‐free survival (PFS), while secondary outcomes included the response rate (RR) and safety profiles. Kaplan–Meier survival curves and Cox proportional hazards models were utilized for data analysis, adjusting for confounding factors such as age, gender, and PD‐L1 expression.

**Results:**

In this study, 160 patients received CP, while 22 received CNI. The CP group was associated with significantly better PFS than the CNI group (median 11.7 vs. 6.6 months, HR 0.56, *p* = 0.03). This PFS advantage persisted after propensity score matching to adjust for imbalances. No significant OS differences were observed. Grade 3–4 adverse events occurred comparably, but immune‐related adverse events were numerically more frequent in the CNI group.

**Conclusions:**

In real‐world practice, CP demonstrated superior PFS compared with CNI. These findings can inform treatment selection in advanced NSCLC.

## Graphical abstract

A retrospective study comparing chemo + pembrolizumab versus chemo + nivolumab + ipilimumab in advanced NSCLC. After propensity score matching, progression‐free survival was significantly longer with chemo + pembrolizumab, while overall survival was comparable between the two regimens.

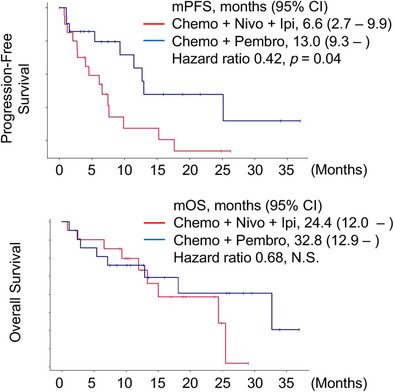

## INTRODUCTION

Lung cancer remains the leading global cause of cancer‐related mortality, with non‐small cell lung cancer (NSCLC) accounting for approximately 85% of cases.^1^ For advanced NSCLC without targetable driver mutations, such as epidermal growth factor receptor (EGFR) or anaplastic lymphoma kinase (ALK), platinum‐doublet chemotherapy has been the standard first‐line treatment for decades, with a median overall survival (OS) of 8–10 months. However, chemotherapy has several limitations, such as toxicity, resistance, and tumor heterogeneity.^2^, ^3^

In recent years, immune checkpoint inhibitors (ICIs) blocking programmed cell death‐1 (PD‐1) and cytotoxic T‐lymphocyte‐associated antigen‐4 (CTLA‐4) have emerged as new therapeutic options in metastatic NSCLC. These agents enhance the antitumor immune response by preventing the interaction between PD‐1 and its ligands (programmed cell death ligand 1: PD‐L1 and programmed cell death ligand 2: PD‐L2) or between CTLA‐4 and its ligands (CD80 and CD86) on the surface of T cells and antigen‐presenting cells, respectively. Multiple phase III trials have shown clinical benefits with ICIs over chemotherapy in the first‐line setting, especially for patients with high PD‐L1 expression.^4^

Based on this evidence, ICIs as monotherapy or combined with chemotherapy are now recommended options for initial therapy in stage IV nonsquamous and squamous NSCLC, regardless of PD‐L1 expression. Two commonly used regimens are: (1) PD‐1 inhibitor (pembrolizumab) plus chemotherapy, and (2) combined PD‐1 inhibitor (nivolumab), CTLA‐4 inhibitor (ipilimumab), and chemotherapy. The KEYNOTE‐189 and KEYNOTE‐407 trials established the pembrolizumab plus chemotherapy regimen in nonsquamous and squamous NSCLC, respectively, showing significant improvements in OS, progression‐free survival (PFS), and response rates over chemotherapy alone.^5^ The CheckMate 9LA trial evaluated nivolumab plus ipilimumab with chemotherapy in patients with any histology and PD‐L1 expression, demonstrating superior OS and PFS over chemotherapy alone.^6^ While both regimens improve survival outcomes over chemotherapy alone, there are no randomized controlled trials directly comparing these regimens. Therefore, there is an unmet need to assess the relative efficacy and safety of these regimens in real‐world practice, where patient characteristics and treatment patterns may differ from those in clinical trials.

This study aimed to compare clinical outcomes and adverse events for pembrolizumab plus chemotherapy versus nivolumab plus ipilimumab plus chemotherapy as first‐line treatments for advanced NSCLC using real‐world data from a multicenter, retrospective cohort of patients in Japan.

## METHODS

### Study design

This was a multicenter, retrospective observational study conducted in collaboration with eight medical facilities located in Kanagawa Prefecture, Japan. The primary objective of this study was to evaluate the efficacy and safety of combined immunotherapy as a first‐line treatment in patients with NSCLC. Ethical approval for the study was granted by the Ethics Committee of Yokohama City University (approval no.: B191200044). Due to the retrospective nature of the study, the requirement for informed consent was waived.

### Study population

The study population included patients diagnosed with NSCLC at any of the eight participating medical facilities between March 1, 2009 and December 31, 2022. Eligible patients were those who received first‐line systemic therapy with chemotherapy and ICIs. Patients were stratified into the CP cohort, treated with cytotoxic agents and pembrolizumab following the KEYNOTE‐189 or 407 protocols^5^, ^7^ and the CNI cohort, receiving cytotoxic agents with ipilimumab and nivolumab as in CheckMate 9LA.^6^

### Assessments

Data on patient demographics, clinical characteristics, treatments, survival outcomes, and adverse events were retrospectively collected from electronic medical records. Survival was defined as the time from initiation of first‐line treatment to date of death from any cause. Patients with unknown vital status were censored at the date of last contact.

Tumor response was assessed by computed tomography (CT) scan based on the response evaluation criteria in solid tumors: Revised RECIST guideline (version 1.1).^8^ Adverse events were graded according to Common Terminology Criteria for Adverse Events (CTCAE) version 5.0.

### Propensity score matching

Propensity scores were estimated for each patient based on a logistic regression model incorporating age, sex, PD‐L1 tumor proportion score (TPS), Eastern Cooperative Oncology Group performance status (ECOG PS), and histology as covariates. Then, patients in the CNI group were matched 1:1 with patients in the CP group using a nearest neighbor matching algorithm and a caliper of 0.2. This yielded 21 matched pairs of patients with balanced baseline demographics.

### Statistical analysis

The baseline characteristics of the patients were summarized and analyzed. Survival outcomes between the cohorts were assessed using Kaplan–Meier estimates and compared with the log‐rank test. Cox proportional hazard regression models were used to calculate hazard ratios and 95% confidence intervals. Differences in objective response rates and disease control rates were evaluated using the Chi‐square test. Statistical analyses were conducted utilizing JMP version 17.0 (SAS Institute Inc., NC, US) and Python version 3.9.17. All tests were two‐tailed, with a significance threshold set at *p* < 0.05.

## RESULTS

### Patient selection and cohort stratification

**FIGURE 1:**
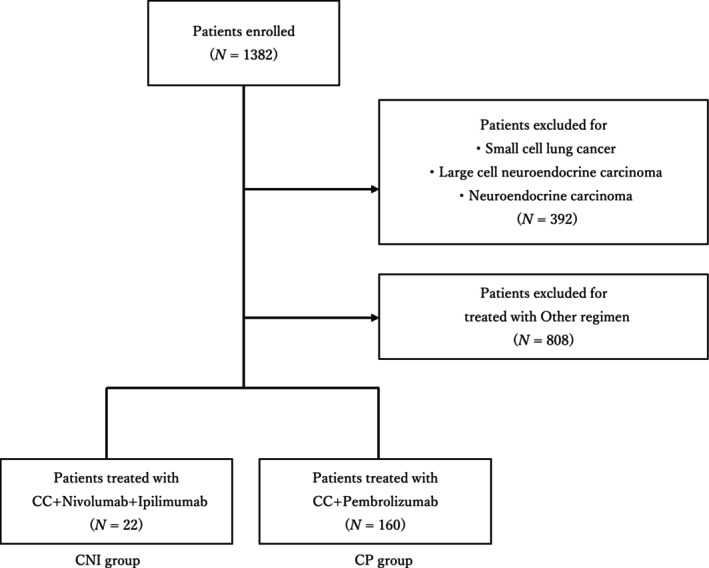
Flow chart of study population allocation into nivolumab plus ipilimumab and chemotherapy (CNI) and pembrolizumab and chemotherapy (CP) groups.

A total of 1382 patients with a diagnosis of primary lung cancer underwent anticancer chemotherapy. From this cohort, 392 patients with small cell lung cancer, large cell neuroendocrine carcinoma, or neuroendocrine tumors were excluded. Furthermore, 808 patients who were treated with regimens other than those specified in the CNI (CheckMate 9LA regimen) or CP (KEYNOTE‐189/ 407 regimen) were also omitted from the analysis. Following these criteria, 22 patients were allocated to the CNI group, and 160 to the CP group (Figure 1).

### Patient characteristics

The baseline characteristics are summarized in Table 1. The median age of patients was 62 years in the CNI group and 69 years in the CP group; the former was significantly younger (*p* = 0.007). The male‐to‐female ratio was about equal in both groups, with males accounting for about 81% of the total number of patients. The distribution of the PD‐L1 TPS was significantly lower in the CNI group (*p* = 0.012). There was no statistically significant difference in the distribution of PS; however, a minority of patients in the CP group exhibited a PS of 3–4. The median follow‐up period was 349 days for the CNI group and 302 days for the CP group.

**TABLE 1:** Patient characteristics.

	CNI	CP	*p*‐value
*N*	22	160	
Age, median (range)	62 (39–78)	69 (40–85)	0.007
Male, *n* (%)	18 (81.8)	131 (81.9)	1.000
Smoking habit, *n* (%)			0.725
Never	2 (9.0)	12 (7.5)	
Former	13 (59.1)	83 (51.9)	
Current	7 (31.8)	63 (39.4)	
Unknown	0	2 (1.2)	
Pathological types, *n* (%)			0.555
Adeno	17 (77.3)	104 (65)	
Squamous	4 (18.2)	42 (26.3)	
Others or unclassifiable	1 (4.5)	14 (8.7)	
Driver oncogene mutations			0.559
None	18 (81.8)	118 (73.8)	
EGFR	1 (4.5)	4 (2.5)	
ALK	0	1 (0.6)	
KRAS	0	12 (7.5)	
MET	0	3 (1.9)	
RET	0	1 (0.6)	
Other	0	1 (0.6)	
Unknown	3 (13.6)	20 (12.5)	
PD‐L1 TPS, *n* (%)			0.012
<1%	12 (54.5)	36 (22.5)	
1%–49%	2 (9.1)	47 (29.4)	
≥50%	5 (22.7)	56 (35)	
Unknown	3 (13.6)	21 (13.1)	
ECOG PS, *n* (%)			0.772
0	7 (31.8)	59 (36.9)	
1	12 (54.5)	85 (53.1)	
2	3 (13.6)	14 (8.8)	
3	0	1 (0.6)	
4	0	1 (0.6)	
Observation period (days), median(range)	349 (23–813)	302 (3–1438)	0.165

Abbreviations: ALK, anaplastic lymphoma kinase; CNI, nivolumab plus ipilimumab and chemotherapy; CP, pembrolizumab and chemotherapy; ECOG PS, Eastern Cooperative Oncology Group performance status; EGFR, epidermal growth factor receptor; KRAS, kirsten rat sarcoma; PD‐L1, programmed cell death ligand 1; TPS, tumor proportion score.

### Outcomes of survival and response to treatment

**FIGURE 2:**
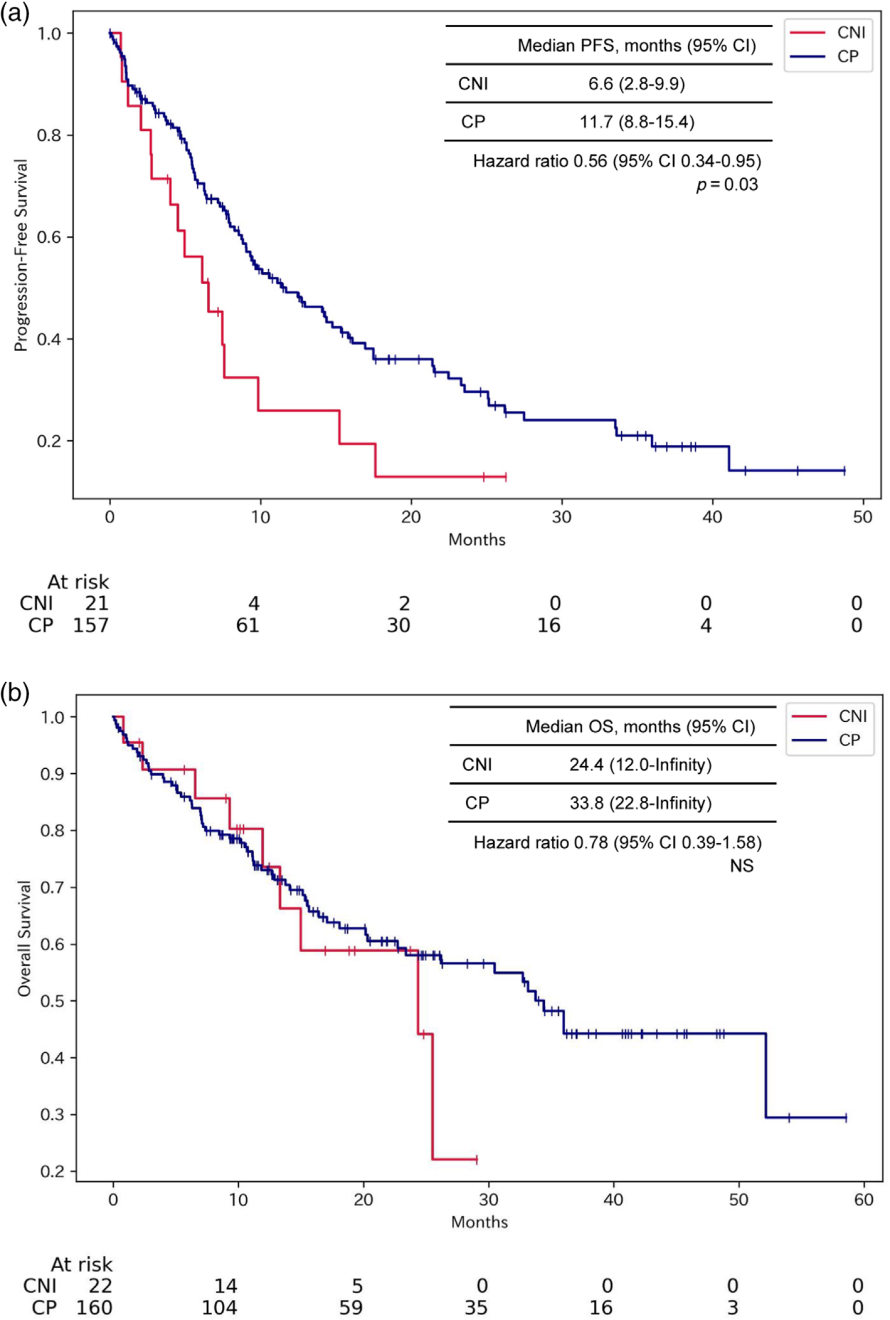
Progression‐free survival (PFS) and overall survival (OS) in CNI versus CP groups. Kaplan–Meier curves comparing PFS and OS between the CNI and CP treatment groups. (a) PFS was significantly longer in the CP group compared with the CNI group (median PFS 11.7 vs. 6.6 months, *p* = 0.03). (b) No significant difference in OS was observed between the CP and CNI groups (median OS 33.8 vs. 24.4 months, *p* = 0.49). The *p*‐values were calculated using the log rank test. CNI, nivolumab plus ipilimumab and chemotherapy; CP, pembrolizumab and chemotherapy.

Kaplan–Meier analysis was used to construct survival curves for PFS (Figure 2a) and OS (Figure 2b). PFS was significantly longer in the CP group compared with the CNI group (11.7 vs. 6.6 months, HR: 0.56, 95% CI: 0.33–0.95, *p* = 0.03). However, differences in OS did not reach statistical significance (median 33.8 vs. 24.4 months; HR: 0.78, 95% CI: 0.39–1.58, *p* = 0.49).

The ORR was comparable between the CNI and CP groups (61.4% vs. 63.1%, *p* = 0.89). The disease control rate (DCR) trended higher in the CP group compared with the CNI group, but the difference was not statistically significant (92.9% vs. 80.0%, *p* = 0.078) (Table 2).

**TABLE 2:** Efficacy outcomes comparing CNI and CP.

	CNI	CP	*p*‐value
CR, *n* (%)	1 (4.5)	10 (6.3)	
PR, *n* (%)	11 (50)	77 (48.1)	
SD, *n* (%)	4 (18.2)	43 (26.9)	
PD, *n* (%)	4 (18.2)	10 (6.3)	
NA, *n* (%)^a^	2 (9.1)	20 (12.5)	
ORR	60.0%	62.1%	1.000
DCR	80.0%	92.9%	0.078

Abbreviations: CNI, nivolumab plus ipilimumab and chemotherapy; CP, pembrolizumab and chemotherapy; CR, complete response; DCR, disease control rate; NA, not applicable; ORR, overall response rate; PD, progressive disease; PR, partial response; SD, stable disease.

^a^ Unknown or died before initial evaluation.

### Adverse events

The incidence of adverse events of any grade is presented in Table 3. Grade 3 or higher adverse events are detailed in Table 4. The rate of grade 3–4 adverse events was comparable between the CNI and CP groups (59.1% vs. 59.4%, *p* = 1.000). Although not statistically significant, immune‐related adverse events (irAEs) numerically occurred more frequently in the CNI group compared with the CP group (36.4% vs. 18.1%, *p* = 0.085). Discontinuation of treatment due to adverse events also tended to be higher in the CNI group relative to the CP group (31.8% vs. 16.9%, *p* = 0.139).

**TABLE 3:** Comparison of the incidence of adverse events in each group.

	CNI	CP	*p*‐value
AE of any grade, *n* (%)	22 (100)	146 (91.3)	
Grade 3 or higher AE, *n* (%)	13 (59.1)	95 (59.4)	1.000
Grade 3 or higher irAE, *n* (%)	8 (36.4)	29 (18.1)	0.085
Discontinuation of treatment due to AE, *n* (%)	7 (31.8)	27 (16.9)	0.139
Death due to serious AE, *n* (%)	2 (9.1)	6 (3.8)	<0.0001

*Note*: Adverse events were graded according to CTCAE version 5.0.

Abbreviations: AE, adverse events; CNI, nivolumab plus ipilimumab and chemotherapy; CP, pembrolizumab and chemotherapy; irAE, immune‐related adverse events.

**TABLE 4:** Severe adverse events in the CNI and CP groups.

	CNI	CP
Bone marrow suppression, *n* (%)	8 (36.4)	61 (38.1)
Adrenal insufficiency, *n* (%)	4 (18.2)	1 (0.6)
Skin rash, *n* (%)	3 (13.6)	3 (1.9)
Renal dysfunction, *n* (%)	3 (13.6)	0
Hepatotoxicity, *n* (%)	1 (4.5)	8 (5.0)
Arthritis, *n* (%)	1 (4.5)	1 (0.6)
Interstitial pneumonia, *n* (%)	1 (4.5)	8 (5.0)
Nausea and vomiting, *n* (%)	0	5 (3.1)
Diarrhea/colitis, *n* (%)	0	4 (2.5)
Fatigue, *n* (%)	0	3 (1.9)
Bacterial infection, *n* (%)	0	2 (1.3)
Hypothyroidism, *n* (%)	0	1 (0.6)
Cholangitis, *n* (%)	0	1 (0.6)
SIADH, *n* (%)	0	1 (0.6)
Pancreatitis, *n* (%)	0	1 (0.6)
Neuropathy, *n* (%)	0	1 (0.6)
Edema, *n* (%)	0	1 (0.6)
Compression fracture, *n* (%)	0	1 (0.6)
Pneumothorax, *n* (%)	0	1 (0.6)
Aspiration, *n* (%)	0	1 (0.6)

*Note*: Adverse events were graded according to CTCAE version 5.0.

Abbreviations: CNI, nivolumab plus ipilimumab and chemotherapy; CP, pembrolizumab and chemotherapy; SIADH, syndrome of inappropriate secretion of antidiuretic hormone.

The most common grade 3–4 adverse events were bone marrow suppression, occurring in over 35% of patients in both groups. In the CNI group, adrenal insufficiency, skin rash, and renal dysfunction were also frequent. Meanwhile, liver dysfunction, interstitial lung disease, and colitis were more prevalent in the CP group.

### Propensity score matching analysis

**FIGURE 3:**
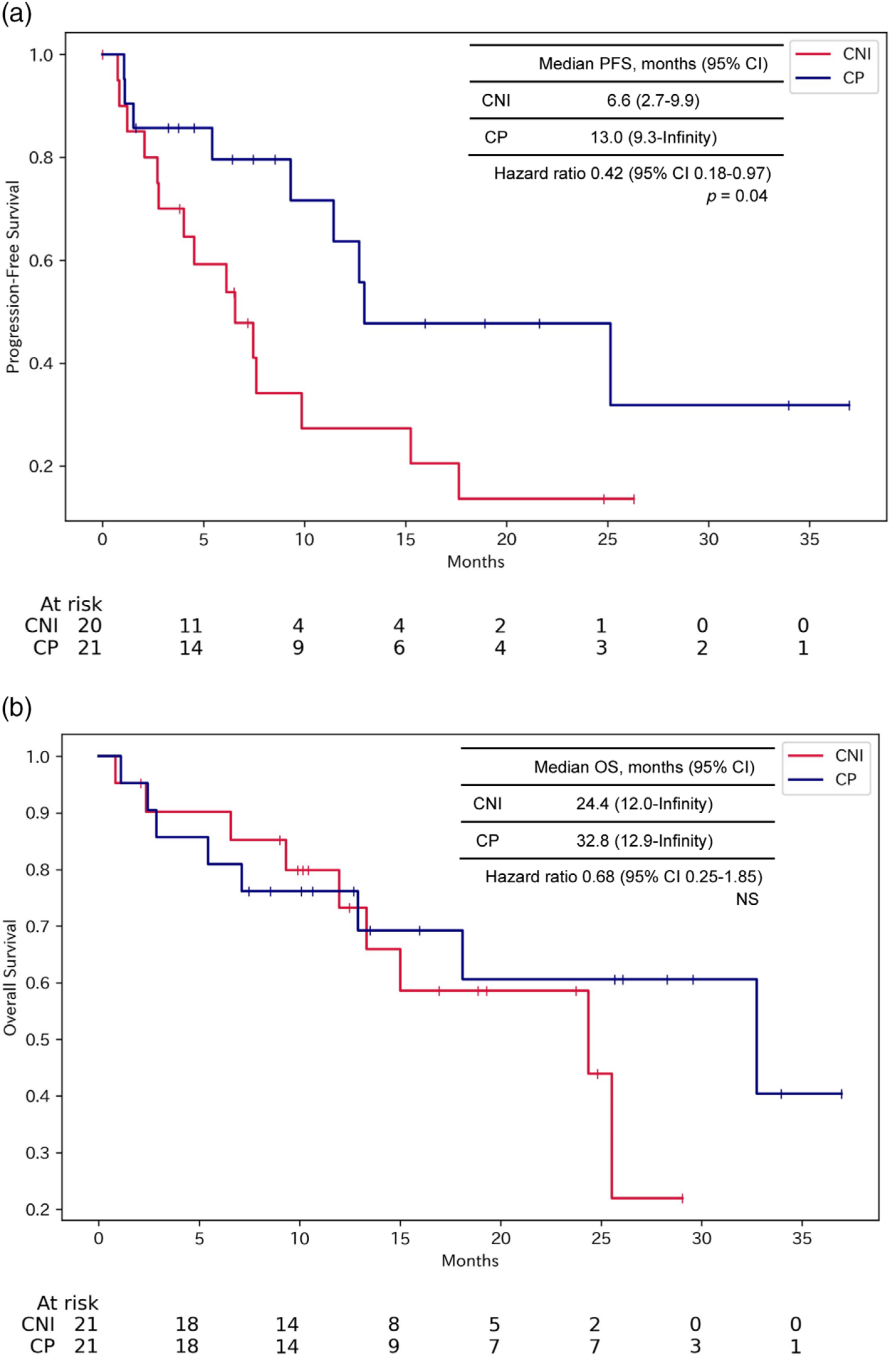
Progression‐free survival (PFS) and overall survival (OS) in propensity score‐matched cohorts. Kaplan–Meier curves for PFS and OS in propensity score‐matched patients treated with CNI or CP regimens. Patients were matched 1:1 based on age, sex, PD‐L1 expression, ECOG performance status, and histology. (a) In the matched analysis, PFS remained significantly longer in the CP group compared with the CNI group (median PFS 13.0 vs. 6.6 months, *p* = 0.04). (b) No significant difference in OS was observed between matched CNI and CP groups (median OS 32.8 vs. 24.4 months, *p* = 0.45). The *p*‐values were calculated using the log rank test. CNI, nivolumab plus ipilimumab and chemotherapy; CP, pembrolizumab and chemotherapy.

Due to substantial imbalance in sample sizes and differences in baseline characteristics between the CNI and CP groups, propensity score matching was conducted to control for potential confounding factors. In the propensity score matched cohort containing 21 patients per group (Table 5), the CP group maintained a statistically significant improvement in PFS compared with the CNI group (median 13.0 months vs. 6.6 months, HR 0.42, 95% CI: 0.18–0.97, *p* = 0.04) (Figure 3a). No significant differences in OS were observed between the matched groups (median 32.8 months vs. 24.4 months, HR 0.68, 95% CI: 0.25–1.85, *p* = 0.45) (Figure 3b).

**TABLE 5:** Patient characteristics after adjusting for propensity score matching.

	CNI	CP	*p*‐value
*n*	21	21	
Age, median (range)	63.6 (50–78)	64.7 (40–79)	0.729
Male, *n* (%)	17 (81.0)	18 (85.7)	1.000
Pathological types			0.111
Adeno	14	19	
Squamous	4	0	
Others or unclassifiable	2	2	
PD‐L1 TPS			0.360
<1%	11	14	
1%–49%	2	3	
≧50%	5	4	
Unknown	3	0	
ECOG PS			0.440
0	6	9	
1	12	11	
2	3	1	
3	0	0	
4	0	0	
Observation period (days), median (range)	349 (23–813)	361 (31–1035)	0.584

Abbreviations: CNI, nivolumab plus ipilimumab and chemotherapy; CP, pembrolizumab and chemotherapy; PD‐L1, programmed cell death ligand 1; ECOG PS, Eastern Cooperative Oncology Group performance status; TPS, tumor proportion score.

### Subgroup analysis by immune‐related adverse events

To investigate the relationship between treatment efficacy and adverse events, subgroups were defined based on the presence or absence of grade 3 or higher irAEs. Kaplan–Meier curves and log‐rank tests were used to compare PFS and OS between the subgroups.

**FIGURE 4:**
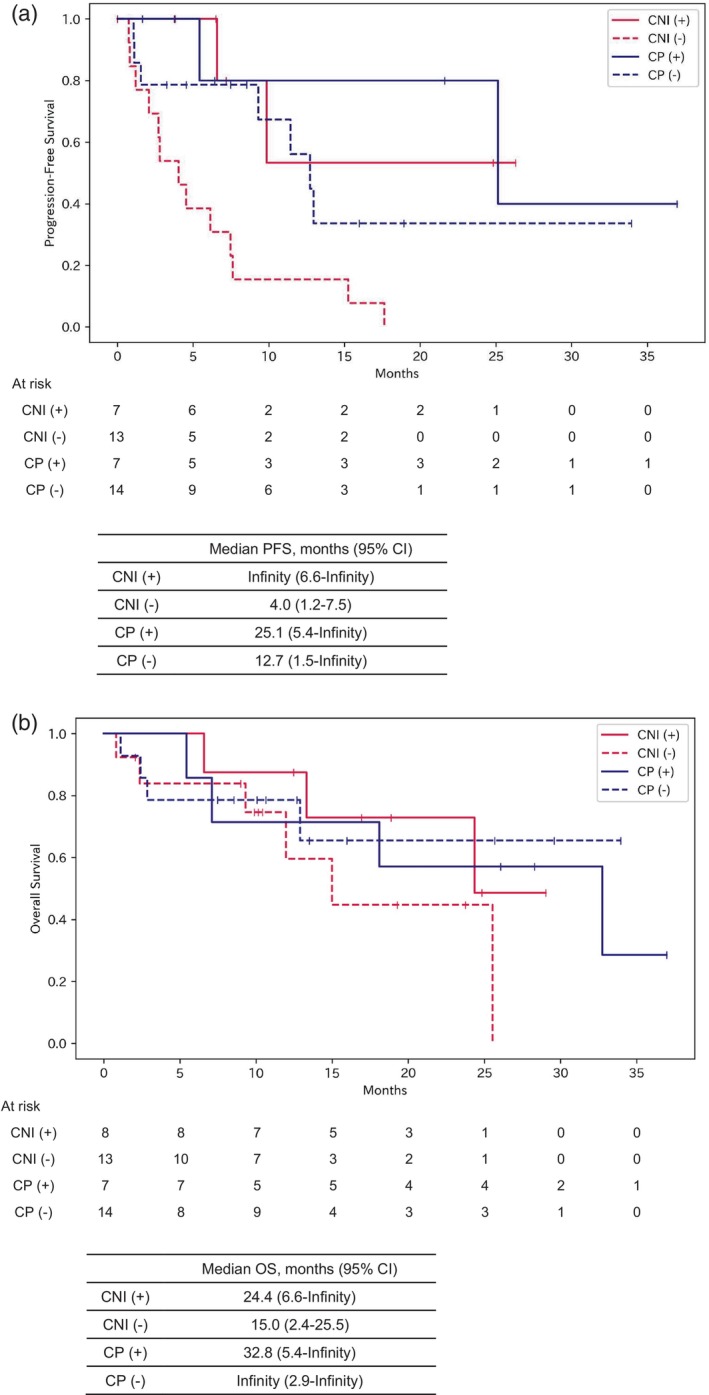
Progression‐free survival (PFS) and overall survival (OS) by immune‐related adverse events. Kaplan–Meier curves for PFS and OS compared between subgroups based on the presence or absence of grade 3 or higher immune‐related adverse events (irAEs). CNI (+) and CP (+) are patients treated with CNI or CP, respectively, who experienced grade ≥3 irAEs. CNI (−) and CP (−) are patients treated with CNI or CP, respectively, with no grade ≥3 irAEs. (a) PFS was significantly shorter in the CNI (−) subgroup compared with the other three subgroups. (b) No significant differences in OS were observed between any subgroups. The *p*‐values were calculated using the log rank test. CNI, nivolumab plus ipilimumab and chemotherapy; CP, pembrolizumab and chemotherapy.

The CNI irAE‐negative subgroup demonstrated significantly shorter PFS compared with the other three subgroups (vs. CP irAE‐positive: *p* = 0.003; vs. CP irAE‐negative: *p* = 0.01; vs. CNI irAE‐positive: *p* = 0.007) (Figure 4a). No significant differences in OS were observed between any subgroups (Figure 4b).

In summary, propensity score matching strengthened the PFS results by adjusting for confounding factors. The subgroup analysis suggested an association between lack of severe irAEs and shorter PFS in the CNI group.

## DISCUSSION

In this retrospective analysis, we compared the efficacy and safety of CNI versus CP as first‐line therapy in patients with recurrent or advanced NSCLC. The results showed that PFS was significantly longer in the CP group (Figures 2a and 3a). However, there was no significant difference in OS between the CNI and CP groups (Figures 2b and 3b). In the safety study, there was a trend toward more serious irAEs and more discontinuations due to adverse events in CNI than in CP (Table 3).

In this study, the PFS, OS, and ORR in the CNI group were 6.6 months, 24.4 months, and 60.0%, respectively. In the CP group, they were 11.7 months, 33.8 months, and 62.1%, respectively (Figure 2 and Table 2). These results can be compared with existing randomized phase 3 trials. The CNI regimen was reported to have a PFS of 6.4 months, OS of 15.6 months, and ORR of 38% in the CheckMate 9LA trial.^6^ It is well‐established that outcomes of drug treatments for NSCLC tend to be more favorable in Japanese patients compared with other ethnicities.^5^, ^7^, ^9^ Hence, the results of the CNI group in this study, which was an observational study including only Japanese patients, should be interpreted with a focus on the PFS being comparable, rather than superior OS and ORR, to the CheckMate 9LA trial. Potential reasons for the shorter PFS with CNI compared with the CP include the significantly higher proportion of patients without PD‐L1 expression in the CNI group in the overall analysis (Figure 2). Additionally, after propensity score matching, there was a higher frequency of squamous cell carcinoma cases in the CNI group, which may confer worse PFS (Figure 3).

Considering histological subtypes, nonsquamous NSCLC typically exhibits higher treatment efficacy and a better prognosis after combination therapy with chemotherapy and ICIs, as reported in the 9LA study.^6^, ^10^, ^11^, ^12^ Notably, the ratio of nonsquamous to squamous cell carcinoma in the 9LA study was 2.1, while our study presented a ratio of 4.5 for CNI and 2.8 for CP (as detailed in Table 1). This disparity favored CNI, yet PFS remained on par with the 9LA study.

As for the CP regimen, separate clinical trials were carried out in nonsquamous (KEYNOTE‐189) and squamous (KEYNOTE‐407) NSCLC histologies. Integrating the outcomes of these trials using the nonsquamous/squamous subtype ratio from the 9LA study yields an adjusted PFS of 8.1 months, OS of 20.1 months, and ORR of 50.7% for the CP regimen. Although a direct comparison is not feasible, these adjusted results and two meta‐analyses suggest a tendency toward superior efficacy with the CP combination compared with CNI, which is in alignment with the findings of the present study.^13^, ^14^

In the current data, a higher frequency of grade 3 or higher adverse events was observed in the CNI group relative to the CP group (Table 3). Previous clinical trials have reported a high incidence of serious adverse events in Japanese populations for both CNI and CP.^7^, ^9^ Currently, a phase 3 trial directly comparing CNI with CP within Japan was prematurely terminated due to a high frequency of serious adverse events in the CNI group.^15^ Although the incidence of irAEs among NI alone in the CheckMate 227 trial did not specifically increase among Asian populations,^16^ the potential for increased serious adverse events with the combination of NI and chemotherapy in East Asian populations should be carefully considered in clinical practice.

CTLA‐4 primarily inhibits T cell activation during the initial stages of immune response, whereas PD‐1 impedes T cells in the later stages of the immune response within peripheral tissues.^17^ Due to these distinct mechanisms, the incidence and characteristics of irAEs differ between anti‐CTLA‐4 and anti‐PD‐1/PD‐L1 antibodies, potentially leading to a higher frequency of irAEs.^13^, ^14^, ^18^, ^19^ However, our data demonstrated an association between irAE occurrence and improved PFS (Figure 4). Several studies, including ours, have reported that irAEs relate to treatment efficacy with ICIs.^20^, ^21^, ^22^ Nonetheless, the irAEs only become evident post‐treatment, rendering them unsuitable as predictive markers for the efficacy of ICI‐containing chemotherapy. Potential biomarkers for clinical efficacy of CTLA‐4 inhibitors and chemotherapy include tumor mutation burden,^23^ T cell receptor coexpression signature, the lung immune prognostic index,^24^ and CTLA‐4 expression on tumors^25^; however, none have been definitively validated. Establishing early predictive biomarkers for CTLA‐4 blockade remains an urgent unmet clinical need.

This study was limited by its retrospective design, small size, lack of randomization, and focus on a single‐country cohort. Retrospective studies can introduce selection bias, and our small number of nivolumab/ipilimumab patients may reduce the ability to detect group differences. Without randomization, propensity matching is still limited and the unequal distribution of prognostic factors cannot be ruled out. Finally, the single‐country population may limit generalizability of the findings to other geographic regions. Further large‐scale prospective studies are needed.

In conclusion, in this real‐world data on advanced NSCLC, pembrolizumab with chemotherapy showed longer PFS and a more favorable side effect profile compared with nivolumab with ipilimumab and chemotherapy. While these findings suggest a preference for the pembrolizumab‐based regimen as initial treatment, further studies with larger cohorts are required.

## AUTHOR CONTRIBUTIONS

**Ayami Kaneko:** Conceptualization, methodology, validation, formal analysis, investigation, data curation, writing‐original draft preparation and visualization. **Nobuaki Kobayashi:** Conceptualization, methodology, validation, formal analysis, investigation, data curation, writing‐review and editing, visualization. **Kenji Miura:** Methodology and data curation. **Hiromi Matsumoto:** Methodology, data curation and visualization. **Kohei Somekawa:** Methodology and data curation. **Tomofumi Hirose:** Data curation. **Yukihito Kajita:** Data curation. **Anna Tanaka:** Data curation. **Shuhei Teranishi:** Data curation and supervision. **Yu Sairenji:** Data curation. **Hidetoshi Kawashima:** Data curation. **Kentaro Yumoto:** Data curation. **Toshinori Tsukahara:** Data curation. **Nobuhiko Fukuda:** Methodology and data curation. **Ryuichi Nishihira:** Data curation and **Keisuke Watanabe:** Investigation. **Nobuyuki Horita:** Supervision. **Yu Hara:** Supervision. **Makoto Kudo:** Supervision. **Naoki Miyazawa:** Data curation. **Takeshi Kaneko:** Supervision. Ayami Kaneko and Nobuaki Kobayashi contributed equally to this work. They are co‐first authors of this article.

## CONFLICT OF INTEREST STATEMENT

No conflicts of interest were declared by the authors.

## Data Availability

The data that support the findings of this study are available on request from the corresponding author. The data are not publicly available due to privacy or ethical restrictions.
